# Expanding the cultivable human archaeome: Methanobrevibacter intestini sp. nov. and strain Methanobrevibacter smithii ‘GRAZ-2’ from human faeces

**DOI:** 10.1099/ijsem.0.006751

**Published:** 2025-04-16

**Authors:** Viktoria Weinberger, Rokhsareh Mohammadzadeh, Marcus Blohs, Kerstin Kalt, Alexander Mahnert, Sarah Moser, Marina Cecovini, Polona Mertelj, Tamara Zurabishvili, Bhawna Arora, Jacqueline Wolf, Tejus Shinde, Tobias Madl, Hansjörg Habisch, Dagmar Kolb, Dominique Pernitsch, Kerstin Hingerl, William Metcalf, Christine Moissl-Eichinger

**Affiliations:** 1 D&R Institute of Hygiene, Microbiology and Environmental Medicine, Medical University of Graz, Graz, Austria; 2 Research Group Metabolomics, Leibniz Institute DSMZ-German Collection of Microorganisms and Cell Cultures GmbH, Braunschweig, Germany; 3 BioTechMed Graz, Graz, Austria; 4 Otto Loewi Research Center, Medicinal Chemistry, Research Unit Integrative Structural Biology, Medical University of Graz, Graz, Austria; 5 Core Facility Ultrastructure Analysis, Medical University of Graz, Graz, Austria; 6 Gottfried Schatz Research Center, Cell Biology, Histology and Embryology, Medical University of Graz, Graz, Austria; 7 Department of Microbiology, University of Illinois, Urbana, Illinois, USA

**Keywords:** faecal methanogens, *Methanobrevibacter intestini*, *Methanobrevibacter smithii*, human archaeome

## Abstract

Two mesophilic, hydrogenotrophic methanogens, WWM1085 and *M. smithii* GRAZ-2, were isolated from human faecal samples. WWM1085 was isolated from an individual in the United States and represents a novel species within the genus *Methanobrevibacter. M. smithii* GRAZ-2 (=DSM 116045) was retrieved from a faecal sample of a European, healthy woman and represents a novel strain within this species. Both *Methanobrevibacter* representatives form non-flagellated, short rods with variable morphologies and the capacity to form filaments. Both isolates showed the typical fluorescence of F_420_ and methane production. Compared to *M. smithii* GRAZ-2, WWM1085 did not accumulate formate when grown with H_2_ and CO_2_. The optimal growth conditions were at 35–39 °C and pH 6.5–7.5. Full genome sequencing revealed a genomic difference of WWM1085 to the type strain of *M. smithii* DSM 861 (=PS^T^), with 93.55% average nucleotide identity (ANI) and major differences in the sequence of its *mcrA* gene (3.3% difference in nucleotide sequence). Differences in the 16S rRNA gene sequence were very minor, and thus distinction based on this gene marker might not be possible. *M. smithii* GRAZ-2 was identified as a novel strain within the species *Methanobrevibacter smithii* (ANI 99.04% to *M. smithii* DSM 861 [=PS^T^]). Due to the major differences between WWM1085 and * M. smithii* type strain *M. smithii* DSM 861 (=PS^T^) in phenotypic, genomic and metabolic features, we propose *Methanobrevibacter intestini* sp. nov. as a novel species with WWM1085 as the type strain (DSM 116060^T^ = CECT 30992^T^).

## Introduction

*Methanobrevibacter* species are widespread and have been found in numerous host microbiomes. They exhibit remarkable adaptability in engaging with both animal hosts and non-archaeal elements within their microbiome. By metabolizing diverse small fermentation by-products, these species effectively facilitate and support various syntrophic interactions. They stand out as the predominant archaea thriving in the gastrointestinal tracts of numerous animals, including humans [1,3].

Among these species, *M. smithii* (with four isolates currently available [4]) represents the most prevalent archaeon within the human gut, exhibiting an average relative abundance of up to 2% in individuals with high methane emission levels in their breath [5].

It is worth noting that the *M. smithii* type strain *M. smithii* DSM 861 (=PS^T^) was initially isolated from sewage samples rather than human faeces [6]. A contamination of the sewage sample with human faeces cannot be excluded, in particular as the gastrointestinal tract is the most favourable habitat for *M. smithii*. In contrast, *M. smithii* DSM 2375 (=ALI) [7] is considered as one of the first publicly available *M. smithii* strains described and isolated directly from human faecal samples.

Additionally, a recent discovery indicated that *M. smithii* encompasses two distinct clades, tentatively labelled as ‘s__Methanobrevibacter_A smithii’ and ‘s__Methanobrevibacter_A smithii_A’ within the GTDB taxonomy [8]. This differentiation was further corroborated through genomic analyses and the incorporation of numerous metagenome-assembled genomes (MAGs) from studies on the human microbiome, confirming the taxonomic separation between s__Methanobrevibacter_A smithii and s__Methanobrevibacter_A smithii_A [9]. It was found that the median genome size of s__Methanobrevibacter_A smithii_A slightly surpasses that of s__Methanobrevibacter_A smithii (1.9 Mbp compared with 1.7 Mbp), while showing an average nucleotide identity (ANI) of 93.95%. Despite this variance, key genes linked to methanogenesis were shared between both strains. The *mcrA* gene exhibited an average amino acid sequence difference of 2.15% [9], a potential marker for distinguishing these clades using molecular methods [10]. Following these observations, s__Methanobrevibacter_A smithii_A was tentatively designated as a distinct species, named *Candidatus* Methanobrevibacter intestini [9].

*Ca*. M. intestini is represented by WWM1085 (formerly recognized as a strain of *M. smithii*), which was initially isolated from human stool in the presence of CO_2_ and H_2_ as a carbon and energy source [9,11]. This species demonstrates extensive distribution and a notably high prevalence among the human population, accounting for approximately 90.01% of all individuals [12]. In the present paper, we further describe *Methanobrevibacter intestini* as a novel species within the *Methanobrevibacter* genus using comparative 16S rRNA and *mcrA* gene as well as full genome sequencing, culture-based methods, electron microscopy, lipidomics and metabolomics. We provide this *Methanobrevibacter intestini* strain WWM1085 as a new addition to the culture collection (DSM 116060=CECT 30992) of anaerobic archaea found in humans. Moreover, we characterize another newly isolated strain of *M. smithii* called *M. smithii* GRAZ-2.

## Methods

### Sources of microorganisms

Strain WWM1085 (DSM 116060 = CECT 30992) was enriched by the Department of Microbiology, University of Illinois, Urbana, IL, USA, from a faecal sample (Mayo Clinic Minnesota, biome number 101159) in the presence of CO_2_ and H_2_ as a carbon and energy source. Further details are provided in the draft genome sequence announcement [11]. The enrichment was subcultured and purified via antibiotic treatment to a pure culture in 2021 at the Medical University of Graz, Austria. In detail, the growth medium (MpT1, see below) was supplemented with streptomycin sulphate (10 mg ml^−1^) and penicillin G potassium salt (10 mg ml^−1^) at a volume ratio of 1 : 100 (0.2 ml of the antibiotics mixture in a volume of 20 ml of medium).

*M. smithii* GRAZ-2 was isolated from a stool sample of a healthy female aged 42 at the Medical University of Graz, Graz, Austria in 2018 in the presence of CO_2_ and H_2_ as a carbon and energy source. This strain is also currently available at DSMZ (=DSM 116045; German Collection of Microorganisms and Cell Cultures GmbH, Braunschweig, Germany).

*M. smithii* DSM 2375 (=ALI) and *M. smithii* DSM 861 (=PS^T^) were obtained from the DSMZ and were used for comparative analysis.

### Ethical approval

Sampling of the human faecal sample was evaluated and approved by the Ethics Committee of the Medical University of Graz (27-151 ex 14/15). Before participation, the participant signed an informed consent.

### Enrichment and isolation of strain GRAZ-2

The stool sample was collected from a fresh faecal sample with an eSwab (COPAN Diagnostics Inc., Italy). The collection fluid, which keeps anaerobic microorganisms alive, was transferred to ATCC medium 1340 (MS medium for methanogens, see below), supplemented with ampicillin (100 µg ml^−1^), streptomycin (100 µg ml^−1^), tetracycline (10 µg ml^−1^) and nystatin (20 µg ml^−1^). Methane production in the culture’s headspace was verified after visible growth (turbidity and microscopy) using a methane sensor (BCP-CH4 sensor, BlueSens, Germany).

Enrichment of methanogens was achieved via fluorescence-activated cell sorting (FACS) exploiting the auto-fluorescence of the cofactor F_420_. FACS was performed at the ZMF Core Facility Molecular Biology in Graz, Austria. For the detection of the F_420_ fluorescence, the violet laser (405 nm) and the bandpass filter 450/40 of the FACSAria III system (Becton Dickinson, USA) were used. During the short sorting process, cells were kept and sorted into reduced medium (1× PBS buffer containing 1 g l^−1^ L(+)-ascorbic acid [13]). 500 000 events were collected and re-grown in liquid MS medium (see below).

Subsequently, the culture was plated on solid MS medium (1.5% agar, w/v) in Hungate tubes using the roll-tube method as described [14]. A single colony was picked and re-grown in liquid medium. To further ensure purity, serial dilutions were performed.

### Growth media

Standard archaeal medium (MS medium) [15] was used to grow all isolates, with some modifications. This medium contained the following constituents (l^−1^ distilled water): 0.45 g NaCl, 5 g NaHCO_3_, 0.1 g MgSO_4_·7H_2_O, 0.225 g KH_2_PO_4_, 0.3 g K_2_HPO_4_·3H_2_O, 0.225 g (NH_4_)_2_SO_4_, 0.060 g CaCl_2_·2H_2_O, 2 ml (NH_4_)_2_Ni(SO_4_)_2_ solution (0.1% w/v), 2 ml FeSO_4_·7 H_2_O solution (0.1% w/v in 0.1 M H_2_SO_4_) and 0.7 ml resazurin solution (0.1% w/v). These compositions were then supplemented with 1 ml of each 10× Wolfe’s vitamin and 10× mineral solutions [15]. The medium was then deoxygenated with N_2_ and 0.75 g l-cysteine was added under anaerobic conditions. pH was adjusted to 7.0 if necessary. 20 ml of liquid was then aliquoted into 100 ml serum bottles, sealed with rubber stopper and aluminium cap and pressurized with H_2_/CO_2_ (4 : 1) before autoclaving. Before use, 1 g l^−1^ of yeast extract and 1 g l^−1^ sodium acetate were added to the medium.

To investigate whether strain WWM1085 could grow using methanol or ethanol as substrates, growth experiments were conducted using modified MS medium under two different gas phases (N_2_ or N_2_/CO_2_) instead of the standard H_2_/CO_2_ gas phase. For methanol experiments, the medium was supplemented with yeast extract (1 g l^−1^), sodium acetate (1.64 g l^−1^), sodium formate (4.08 g l^−1^) and methanol (200 mM). For the experiments with ethanol, the medium was likewise supplemented with yeast extract, sodium acetate and sodium formate and 400 mM ethanol. Methanol and ethanol levels were chosen according to Li *et al.* [16].

For the growth of WWM1085, MpT1 medium, based on AM-5 [17], was used with some modifications. Modified MpT1 medium had the following compositions (l^−1^ distilled water): 1 g NaCl, 0.5 g KCl, 0.19 g MgCl_2_, 0.1 g CaCl_2_·2H_2_O, 0.3 g NH_4_Cl, 0.2 g KH_2_PO_4_, 0.15 g Na_2_SO_4_, 2 g casamino acids, 2 g yeast extract and 0.082 g sodium acetate. Then, 1 ml trace element solution (1.2 ml HCl [12.5 M], 0.01 g MnCl_2_·4H_2_O, 0.019 g CoCl_2_·6H_2_O, 0.0144 g ZnSO_4_·7H_2_O, 0.0002 g CuCl_2_·2H_2_O, 0.003 g H_3_BO_3_, 0.0024 g NiCl_2_·6H_2_O, 0.0036 g Na_2_MoO_4_·2H_2_O in 150 ml distilled water), 20 µl of selenite–tungstate solution (2 g NaOH, 0.01 g Na_2_SeO_3_5H_2_O and 0.017 g Na_2_WO_4_·2 H_2_O dissolved in 50 ml distilled water) and 0.7 ml resazurin solution (0.1% w/v) were added. The medium was deoxygenated with N_2_ and subsequently, 0.24 g l-cysteine and 2.52 g NaHCO_3_ were added. 20 ml of medium was distributed in 100 ml serum bottle and was then sealed and pressurized with H_2_/CO_2_ (4 : 1). After autoclaving, 0.2 ml of the following were added to each bottle under anoxic conditions: methanol (50 mM), dithiothreitol (0.154 g l^−1^), Na-formate (0.034 g l^−1^), Na-coenzyme M (0.01 g l^−1^) and vitamin solution (3 mg biotin, 3 mg folic acid, 15 mg vitamin B6, 7.5 mg vitamin B1, 7.5 mg vitamin B2, 7.5 mg nicotinic acid, 7.5 mg d,l-panthothenic acid, 1.5 mg vitamin B12, 7.5 mg *p*-aminobenzoic acid, 0.3 g choline chloride dissolved in 150 ml distilled water). The pH was adjusted to 7.0 if applicable.

All growth experiments were carried out in triplicates under static conditions at 37 °C unless mentioned otherwise.

### Scanning electron microscopy

For scanning electron microscopy (SEM), cells were mounted on coverslips, fixed with 2 % (w/v) paraformaldehyde in 0.1 M phosphate-buffered saline (PBS), pH 7.4 and 2.5% glutaraldehyde in 0.1 M PBS, pH 7.4 and dehydrated stepwise in a graded ethanol series. Samples were post-fixed with 1% osmium tetroxide for 1 h at room temperature and subsequently dehydrated in graded ethanol series (30–96% and 100% (v/v) EtOH). Further, hexamethyldisilane (HMDS Merck, Sigma-Aldrich, USA) was applied. Coverslips were placed on stubs covered with a conductive double-coated carbon tape. The images were taken with a Sigma 500VP FE-SEM with a SEM Detector (Zeiss Oberkochen, Germany) operated at an acceleration voltage of 5 kV.

### Optimum pH and temperature

The 100 ml serum bottles, each containing 20 ml of modified standard archaeal medium (for all tested isolates) and MpT1 (only for WWM1085), were inoculated with 2.5% (v/v) fresh cultures. Growth (measured in terms of OD at 600 nm) and methane production were monitored daily for 10 days to assess the impact of pH and temperature on growth. Methane levels were quantified using a gas sensor (BCP-CH4 sensor, BlueSens, Germany), and data integration and analysis were performed using the provided BacVis Gas Formation software.

To explore the effect of pH on growth, media with different pH values ranging from 5 to 11 were prepared by adjusting with varying amounts of 0.1 M NaOH or 0.1 M HCl. The pH values of the media were checked daily for potential alterations (pH indicator strips, VWR, Germany) and were maintained constant. The optimum pH was determined at 37 °C.

For the determination of the optimum temperature, cultures were incubated at various temperatures (20, 30, 35, 37, 39, 40 and 50 °C), while pH was kept constant at 7. Temperature was monitored continuously using a temperature logger (Sensor Blue, Brifit) inside the incubator. Both pH and temperature experiments for WWM1085 were conducted in modified standard archaeal medium and MpT1.

### Culture purity check and sequencing

Cultures were routinely checked for purity using microscopy, PCR and Sanger sequencing. Microscopic examination of the cells was performed using a Nikon microscope equipped with a fluorescence attachment and a UV excitation filter. Extracted DNA was subjected to PCR targeting the archaeal *mcrA* (forward primer sequence: 5′-CAACCCAGACATTGGTACTCCT-3′, reverse: 5′-GCTGGGGTGATGACAGTTCT-3′) and the bacterial 16S rRNA gene (primers 341 F and 1391 R) [18,19]. Media blanks and no-template controls served as negative controls.

### Nanopore sequencing

*M. smithii* GRAZ-2 underwent Nanopore sequencing using the MinION Mk1C system (Oxford Nanopore Technologies plc., United Kingdom) according to the protocols as detailed (nanoporetech.com). To summarize, DNA extraction was done according to the manufacturer’s protocol (Invitrogen^TM^ PureLink^TM^ Microbiome DNA Purification Kit, Thermo Fisher Scientific Inc, USA). Subsequently, the Nanodrop 2000c spectrophotometer (Thermo Fisher Scientific Inc., USA) and an Invitrogen^TM^ Qubit^TM^ 3 Fluorometer (Thermo Fisher Scientific Inc., USA) were used to confirm the quality and concentration of the extracted DNA. In addition, gel electrophoresis was used for checking DNA fragmentation. DNA was then stored at −20 °C for further analyses.

In the process of preparing the library, DNA underwent repair utilizing the NEBNext Companion Module (New England Biolabs GmbH, Germany). Subsequently, it was prepared for sequencing on a chemistry version 14 flow cell (R10.4.1, FLO-MIN114) following the Ligation sequencing gDNA – Native Barcoding Kit 24 V14 (SQK-NBD114.24) as outlined by the guidelines of the manufacturer as detailed in nanoporetech.com.

### DNA-based comparisons

#### 16S rRNA genes

The 16S rRNA genes of type strain representatives of the *Methanobrevibacter* genus and *M. smithii* strains were retrieved through NCBI (accession numbers are provided in the tree and Table S1, available in the online version of this article). For *Methanobrevibacter smithii* DSM 2374 (=F1), no Sanger sequence was available, and instead, the 16S rRNA gene was extracted from its full genome information (accession number and genomic location provided in Table S1).

Alignment of the sequences was performed via Muscle [20,21], implemented in mega11 (standard settings of mega11 [22]). All aligned 16S rRNA genes were manually trimmed to the same length. Pairwise distance estimation was performed using the standard settings. The matrix is available in Table S1. For the Maximum Likelihood tree, the alignment was subjected to tree calculation (bootstrap alignments: 1000) via the W-IQ-TREE web server (standard settings) [23].

#### mcrA genes

*mcrA* genes were retrieved through MAGE genoscope ([24]; Table S2), a platform for genomic comparison. Genes were aligned using Muscle (see above), and pairwise distance estimation was calculated using the standard settings of mega11. The matrix is provided in Table S3.

#### Genomic identification of a novel species

The probability of whether one or two isolated *Methanobrevibacter* strains represent novel species was tested using JSpeciesWS [25]. The ANI was calculated against all isolates listed in Table S4, and those provided by the included, curated reference database GenomesDB. This tool also provided the G+C content of each genome. Digital DNA–DNA hybridization was performed using the DSMZ TYGS tool [26]. The average amino acid identity (AAI) was determined by using the AAI calculator (http://enve-omics.ce.gatech.edu/aai/ [27], after downloading the representative protein fasta files from the MAGE genoscope.

#### Genome trees

The genome tree (genus *Methanobrevibacter*) was inferred using GTDB-Tk [28], using the de_novo_wf function. Taxa were filtered for the family *Methanobacteriaceae*, and all non-*Methanobrevibacter* clades were used as outgroup taxon; all other settings were default.

### Lipid and carbohydrate profile analyses by mass spectrometry

Intact polar lipids were extracted from freeze-dried material (approx. 30 mg) using a modified Bligh and Dyer extraction as described previously [29,32]. Briefly, two extractions each were performed using methanol/dichloromethane (DCM)/50 mM phosphate buffer pH 7–8 (2 : 1 : 0.8; v/v/v) and methanol/DCM/0.3 M trichloroacetic acid pH 2–3 (2 : 1 : 0.8 v/v/v). Combined supernatants were adjusted to a ratio of methanol/DCM/50 mM phosphate buffer of 2 : 1 : 0.9 (v/v/v) by adding DCM and phosphate buffer, before the DCM phase was collected. The remaining mixture was additionally extracted twice with DCM, and the combined DCM phases were evaporated to dryness. For HPLC-MS/MS analysis, dried extracts were recovered in hexane/isopropanol/water (718 : 271 : 10; v/v/v).

Archaeal lipids were separated on a YMC-Triart Diol column (150×2.0 mm, 1.9 µm particles) and analysed in positive ESI mode by mass spectrometry on an Agilent 6545 Q-TOF mass spectrometer (Agilent, Waldbronn, Germany) as described previously [29,33]. Mass spectra were recorded in the mass range of *m*/*z* 300–2000. Core lipids were identified by the exact masses of their [M + H]^+^ ions.

For analysis of lipid-associated sugars, lipid extracts were prepared as described above and hydrolyzed according to [34] with slight modifications. Briefly, dried extracts were dissolved in 1 ml 2 N H_2_SO_4_ and incubated for 2 h at 100 °C. Afterwards, the samples were chilled on ice and neutralized by adding 2 N NaOH (final pH 6–8). After centrifugation, the supernatant was evaporated to dryness.

For GC-MS analysis of sugar residues, dried extracts were reconstituted in 1 ml methanol and filtered through a Nylon spin filter to remove excess salt. The remaining supernatant was mixed with 10 µl of a 4 % ribitol–methanol solution and dried under a stream of nitrogen. In addition, non-hydrolyzed extracts were analysed to detect any residual free sugars in the lipid extracts. Derivatization and GC-MS analysis were performed as described previously [35]. Data analysis was performed with the MetaboliteDetector software [36] as described previously [37].

### Quantification of metabolic activity by NMR spectroscopy

A minimum of three replicates for each of the studied archaeal cultures (WWM1085, *M. smithii* GRAZ-2, *M. smithii* DSM 2375 [=ALI]) at different time points after inoculation (72, 168 and 240h) were subjected to analysis, utilizing nuclear magnetic resonance (NMR) spectroscopy, following the methodology outlined before [5]. Briefly, a methanol–water mixture (2 : 1) was used to eliminate proteins from samples followed by centrifugation. Subsequently, the supernatant was lyophilized, re-dissolved in NMR buffer in D_2_O (0.08 M Na_2_HPO_4_, 5 mM 3-(trimethylsilyl) propionic acid-2,2,3,3-d_4_ sodium salt [TSP], 0.04 (w/v) % NaN_3_ in D_2_O, pH adjusted to 7.4 with 8 M HCl and 5 M NaOH) and subsequently transferred to NMR tubes. NMR analysis was then conducted on a Bruker Avance Neo NMR spectrometer running at 600 MHz and equipped with a TXI probe head at 310 K and Topsin 4.3 software (Bruker GmbH, Rheinstetten, Germany). The obtained spectra (cmpgpr1d/Carr–Purcell–Meiboom–Gill pulse sequence with 128 scans) were further processed using MATLAB 2014b (Mathworks, Natick, MA, USA), aligned and normalized by probabilistic quotient normalization [38,39]. For absolute quantification of carbonic acids, known peaks of substances of aligned raw spectra were integrated using trapezium subtraction for baseline correction [40], and eventually normalized on their respective proton number, J-coupling pattern and TMSP integral of the sample to calculate their molar concentrations.

## Results and discussion

Based on our findings, the investigated archaeal strains, namely WWM1085 and *M. smithii* GRAZ-2, along with *M. smithii* DSM 861 (=PS^T^), and *M. smithii* DSM 2375 (=ALI) which were used for comparison, exhibit unique features discerned through our culture-based, genomics and metabolomics methods. Their characteristics are outlined in Tables1  2 and elaborated upon in the subsequent sections.

**Table 1.** Optimal pH and temperature for the growth of the studied strains. Growth was determined by measuring OD_600_ in the growth medium and the ability of the strains to produce methane. (+) minimal growth; (++) moderate growth; (+++) optimal growth; (−) no growth; ND: not determined

Strain		*M. smithii* DSM 861 (=PS^T^)	*M. smithii* DSM 2375 (=ALI)	*M. smithii* GRAZ-2	WWM1085	
**Growth condition**	**Medium**	MS	MS	MS	MS	Modified MpT1
**Temperature(°C)**	20	−	−	−	−	−
	30	−	−	+	++	++
	35	++	−	+	+++	++
	37	+++	++	++	+++	+++
	39	++	+++	+++	+++	+++
	40	+	+++	+++	+	++
	50	−	−	−	−	−
**pH**	5	−	−	−	−	−
	5.5	−	+	+	+	nd
	6	−	+	+	+	nd
	6.5	+++	+	+	++	+++
	7	+++	+++	+++	+++	++
	7.5	++	+++	+++	+++	++
	8	++	++	++	++	++
	9	+	++	++	++	+
	10	−	++	+	+	+
	11	−	−	−	−	−

**Table 2.** Morphological, genomic and physiological features of the strains *M. smithii* DSM 861 (=PS^T^), *M. smithii* DSM 2375 (=ALI)*, M. smithii* GRAZ-2 and WWM1085. Cell size information for type strain *M. smithii* DSM 861 (=PS^T^) taken from [43]. Med: medium

Trait	*M. smithii* DSM 861 (=PS^T^)	*M. smithii* DSM 2375 (=ALI)	*M. smithii* GRAZ-2	WWM1085 (MS med.)	WWM1085 (MpT1 med.)
**DSM catalogue number**	DSM 861	DSM 2375	DSM 116045	DSM 116060	
**Cell shape**	Short, lancet-shaped to oval cocci	Coccobacillus, short rods	Coccobacillus, short rods	Coccobacillus, short rods	
**Cell size, width, length**	0.5–1.0 µm1.0–1.5 µm	0.22–0.54 µm0.26–0.77 µm	0.18–0.58 µm0.33–1.46 µm	0.16–0.43 µm0.29–0.54 µm	
**Genome size**	1.85 Mbp	1.71 Mbp	1.79 Mbp	1.9 Mbp	
**DNA G + C content (mol%)**	31.03	31.28	31.11	30.30	
**N50**	1.85 Mbp	226.2 kb	1.79 Mbp	240.4 kb	
**Number of contigs**	1	24	1	16	
**Number of CDS**	1838	1712	1906	1875	
**Number of tRNAs**	34	33	34	34	
**Lipid profile**	Mostly archaeol	Mostly archaeol	Mostly archaeol	Mostly (cald-)archaeol	
**Temperature range (°C)**	35–40	37–40	30–40	30–40	
**Optimal temperature (°C)**	37	39–40	39–40	35–39	37–39
**pH range**	6.5–9	5.5–10	5.5–10	5.5–10	6.5–10
**Optimal pH**	6.5–7	7–7.5	7–7.5	7–7.5	6.5
**Growth on formate as sole electron source**	No	No	No	No	
**Growth on ethanol/methanol as H_2_ substitute**	No/No	No/No	No/No	No/No	
**CO_2_/H_2_ as carbon and energy source**	Yes	Yes	Yes	Yes	
**Isolation source**	Sludge	Human	Human	Human	

### Morphology

**Fig. 1.**
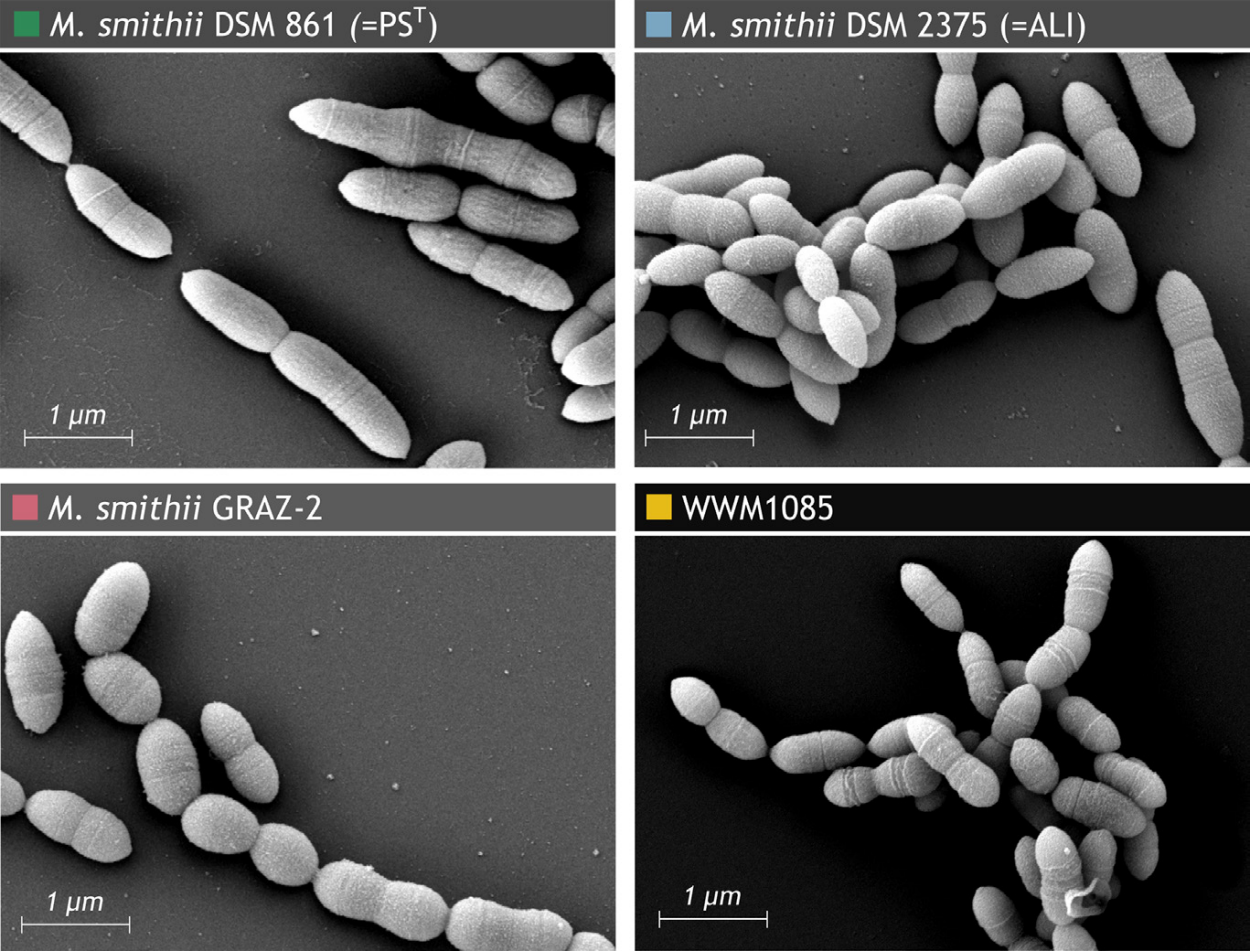
Scanning electron micrograph of *Methanobrevibacter smithii* DSM 861 (=PS^T^)*, Methanobrevibacter smithii* DSM 2375 (=ALI), *Methanobrevibacter smithii* GRAZ-2 and WWM1085.

WWM1085 cells appeared morphologically similar to all other *Methanobrevibacter* species and strains, albeit being slightly shorter. In general, they measure 0.16–0.43 µm in width and 0.29–0.54 µm in length and appear mostly in the form of short rods with rounded ends (Fig. 1; for *M. smithii* DSM 861 [=PS^T^] morphology, see also [41,42]). Similar to the *M. smithii* strains, not only did they occur in single cells, but they were also observed more frequently in pairs, short chains or long filaments. Pili or flagella were not detected, but some cells appeared fluffy on their surface. All isolates tested showed F_420_ fluorescence, which is typical for methanogenic archaea, when observed under fluorescence microscopy (excitation 420 nm). No cells were observed in media controls.

### Substrates and nutritional requirements

WWM1085 underwent growth testing in two media (MS and MpT1), with various substrates (acetate and yeast extract, formate, ethanol and methanol) to assess potential variations in its nutritional requirements compared to *M. smithii* DSM 861 (=PS^T^), * M. smithii* DSM 2375 (=ALI) and *M. smithii* GRAZ-2. At pH 7 and 37 °C, this strain demonstrated optimal growth in both media and reached high cell density after 72 h (2.5% (v/v) inoculation), utilizing H_2_/CO_2_ as its sole energy source. No growth was observed when growth media were exposed to oxygen. Further, no growth on formate, ethanol and methanol as carbon/electron donors was observed.

### Optimum pH range for growth and methane production

WWM1085 (in both media)*,* along with *M. smithii* DSM 861 (=PS^T^), *M. smithii* DSM 2375 (=ALI) and *M. smithii* GRAZ-2 constantly produced methane across a broad pH spectrum (5–11) (Table 1). Based on methane production and OD_600_ measurements, the optimum pH for WWM1085 was found to be 7–7.5 in the MS medium. In modified MpT1, WWM1085 showed the optimal growth at a pH of 6.5. The type strain *M. smithii* DSM 861 (=PS^T^) showed an optimal pH range between pH 6.9 and 7.4 [43], which could be confirmed herein (Table 1). None of the isolates showed growth at pH 5 and 11.

### Optimum temperature range for growth and methane production

All four isolates exhibited growth and methane production within a temperature range of 30–40 °C (30, 35, 37, 39 and 40 °C; Table 1). WWM1085 displayed a broader but lower temperature range for moderate or optimal growth (35–39 °C) as compared with the other three isolates. A similar optimal temperature was observed in the modified MpT1 medium (37–39 °C). The type strain *M. smithii* DSM 861 (=PS^T^) showed an optimal temperature of around 37 °C, which is in agreement with the original description (37–39 °C) [43]. No growth was observed under more extreme temperature conditions (20 °C or 50 °C) for any of the isolates.

### Phylogenetic relationships

**Fig. 2.**
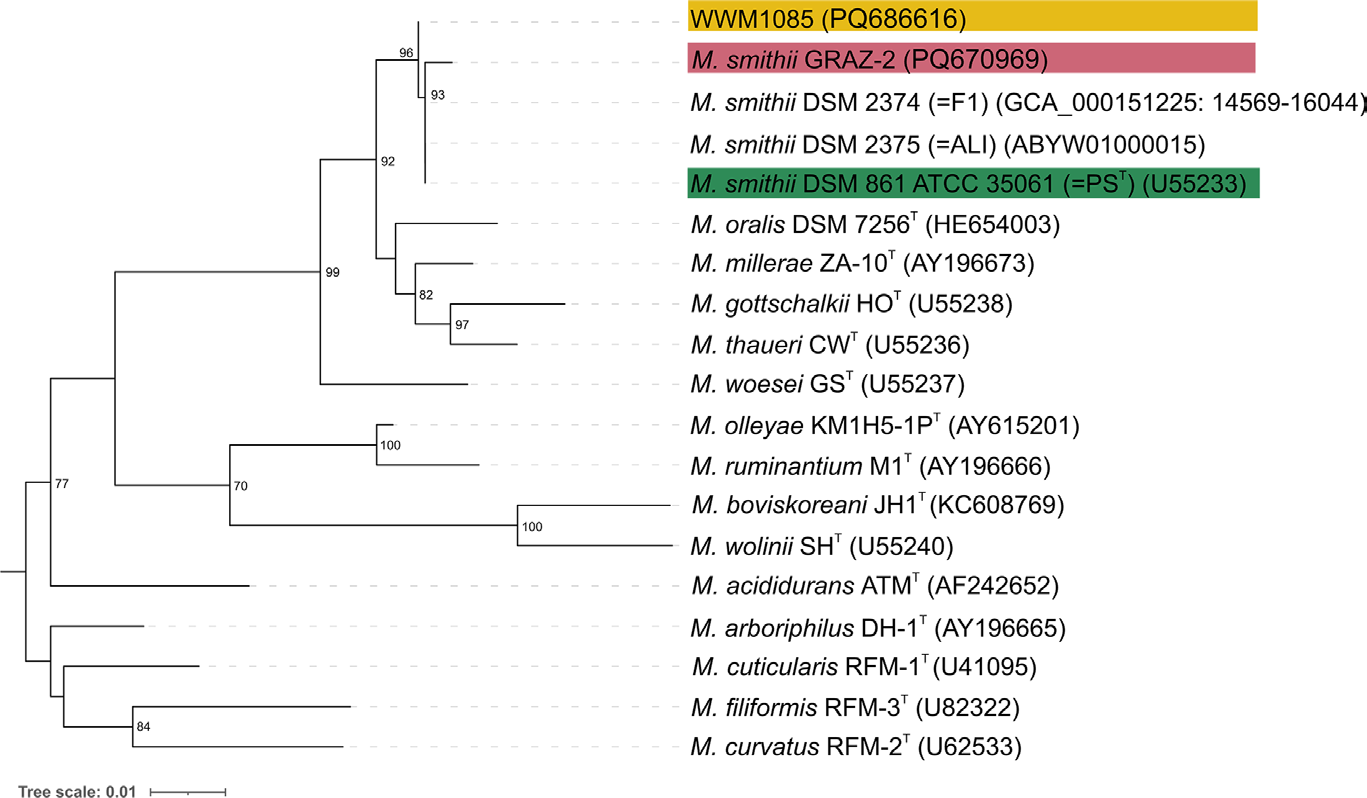
Phylogenetic relationship (Maximum Likelihood tree) of the *Methanobrevibacter* isolates (WWM1085 in yellow, *M. smithii* DSM 2375 [=ALI] in blue, *M. smithii* GRAZ-2 in pink, *M. smithii* DSM 861 [=PS^T^] in green) based on 16S rRNA gene sequence analysis. Accession numbers of Sanger-sequenced 16S rRNA genes are provided in brackets; in case the 16S rRNA gene had to be retrieved from a genomic sequence, the location is provided as well. The tree was inferred by using the Maximum Likelihood method, and the consensus tree is shown here, including the bootstrap values (70 or higher) obtained from 1000 iterations.

The full-length 16S rRNA gene analysis of WWM1085 showed only small variations as compared to closely related *Methanobrevibacter smithii* isolates (*M. smithii* DSM 861 (=PS^T^) and *M. smithii* DSM 2375 (=ALI): 0.046% sequence dissimilarity; Table S1). These only slight discrepancies pose a challenge for differentiating the isolates solely through 16S rRNA gene sequencing. The 16S rRNA gene of *M. smithii* GRAZ-2 was found to be highly similar to the genes of *M. smithii* DSM 861 (=PS^T^) and *M. smithii* DSM 2375 (=ALI) (difference: 0.186 %; Table S1) (Fig. 2).

Differences were more pronounced at the *mcrA* gene level. At the nucleotide level, a minimum of 3% difference was observed between WWM1085 and *M. smithii* DSM 2374 (=F1), *M. smithii* DSM 2375 (=ALI) and *M. smithii* DSM 861 (=PS^T^). On the other hand, *M. smithii* GRAZ-2 showed a difference of 0.0604–0.242% to the aforementioned strains, and therefore showed a higher similarity (Tables S2 and S3). On amino acid level, the *mcrA* gene of WWM1085 showed a difference of 0.0703% to * M. smithii* DSM 2374 (=F1).

When performing pairwise ANI calculations, the similarity values of WWM1085 against strains from GenomesDB and the culture collection consistently fell well below the species threshold (cutoff: 95%). The closest relatives were found to be *M. smithii* DSM 861 (=PS^T^) (ANI: 93.55%) and *M. smithii* DSM 2375 (=ALI) (ANI: 93.04%) (the ANI-matrix is provided in Table S4). Notably, WWM1085 exhibited a slightly lower G+C content as compared to *M. smithii* strains from the DSMZ collection (30.3% as opposed to 31.0–31.3 %) (Table 2). Digital DNA–DNA hybridization [26] revealed a dDDH value of 51.1% [CI 48.4–53.7] for *M. smithii* DSM 861 (=PS^T^) versus WWM1085, which is well below the species threshold of 70% [44]. The two-way AAI for *M. smithii* DSM 861 (=PS^T^) versus WWM1085 (based on 1605 shared proteins) was identified to be 94.50%, which is below the anticipated species threshold of 95% [45].

Consequently, it can be concluded that WWM1085 represents a distinct species within the *Methanobrevibacter* genus. However, it is important to note that despite these genomic differences, the disparities in the 16S rRNA gene are subtle, and in some cases, imperceptible in amplicon-based studies.

**Fig. 3.**
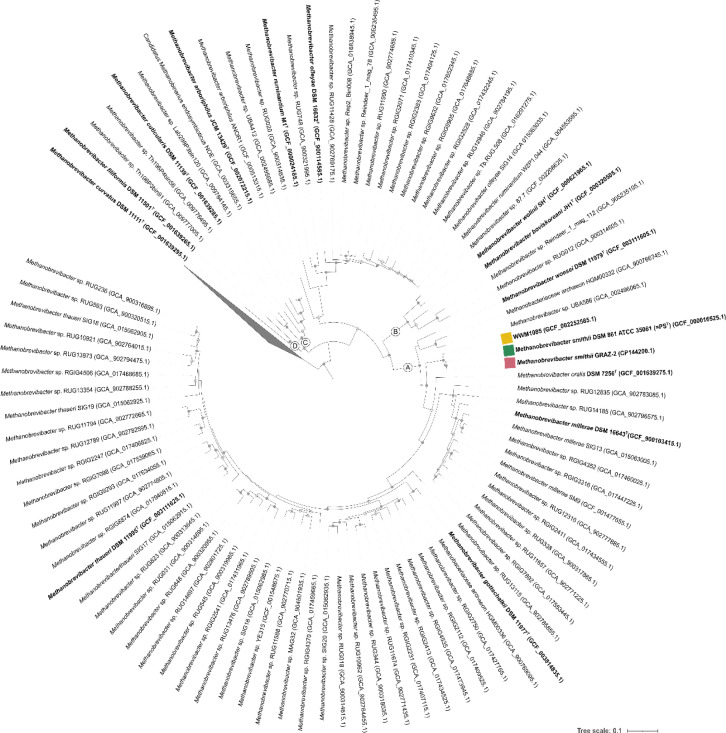
Genome tree of the *Methanobacteriaceae* family based on all GTDB species representatives. Pink square indicates *M. smithii* GRAZ-2, green square indicates *M. smithii* DSM 861 (=PS^T^) and yellow square represents WWM1085. Alignments were formed from the concatenation of 53 (arc53) phylogenetically informative markers, as implemented in the GTDBtk [28] toolkit. Type strains and new isolates are highlighted in bold. *Methanobrevibacter* clades A–D are indicated at the respective branches. Nodes of WWM1085, *M. smithii* DSM 861 (=PS^T^) and *M. smithii* DSM 2375 (=ALI) are supported by a bootstrap value of 1, which are indicated by grey dots; bootstrap values between 0.9 and 1 are shown by grey circles. The collapsed fraction represents the outgroup (Non-*Methanobrevibacter* Methanobacteriales).

*M. smithii* GRAZ-2 showed the closest relationship to *M. smithii* DSM 861 (=PS^T^) (ANI: 99.04%) and therefore does not represent a novel species within the *Methanobrevibacter* genus (Table S4). For visualization, a genome-based tree is provided in Fig. 3.

### Polar lipid composition and lipid-associated sugars

Major detected lipids were largely congruent across species and strains, with archaeol (C_43_H_88_O_3_) being the most prevalent lipid (relative abundance for *M. smithii* DSM 861 (=PS^T^): 96.08%, *M. smithii* DSM 2375 (=ALI): 83.93%; WWM1085 : 72.57%; and *M. smithii* GRAZ-2 : 93.85%), followed by caldarchaeol (C_86_H_172_O_6_) (*M. smithii* DSM 861 (=PS^T^): 2.95%, *M. smithii* DSM 2375 (=ALI): 13.65%; WWM1085 : 26.37%; *M. smithii* GRAZ-2 : 5.81%) and cyclic archaeol (C_43_H_86_O_3_) (M. *smithii* DSM 861 (=PS^T^): 0.97%, *M. smithii* DSM 2375 (=ALI): 0.65%; WWM1085 : 1.06%; *M. smithii* GRAZ-2 : 0.34%). Traces of glycerol dialkyl glycerol tetraether lipids (GDGT-1) or H-shaped caldarchaeol were found in *M. smithii* DSM 2375 (=ALI) (1.77%), but not in the other strains.

Lipid-associated sugar profiles were very similar for all strains, with glucose being most prevalent, accompanied by minor amounts of fructose, rhamnose, ribose and xylose.

### Comparative genomics and metabolomics

Detailed genomic comparisons of WWM1085 with available *M. smithii* genomes are provided in our earlier publication [9], indicating several differences. For instance, WWM1085 does not possess modA/B for molybdate transport. The s__Methanobrevibacter_A_smithii_A (including those genomes from MAGs) were further characterized by additional unique membrane/cell wall-associated proteins, such as adhesin-like proteins, surface proteins and a number of uncharacterized membrane proteins/transporters [9]. Notably, *M. smithii* GRAZ-2 and the WWM1085 genome contained the ABC.FEV.P/S/A iron transport system (EC 3.6.3.34), which was distinctive to all other tested genomes, indicating a potential adaptation towards the human gut environment, where iron is a highly demanded resource.

Utilizing NMR-based metabolomics, the turnover of metabolites was examined among three strains (*M. smithii* DSM 2375 (=ALI), *M. smithii* GRAZ-2 and WWM1085) in MS medium containing yeast extract. All strains reached the stationary phase after 72 h (*M. smithii* DSM 2375 (=ALI) and WWM1085) or at the latest, after 168 h (*M. smithii* GRAZ-2) of growth (growth curves shown in Fig. S1).

**Fig. 4.**
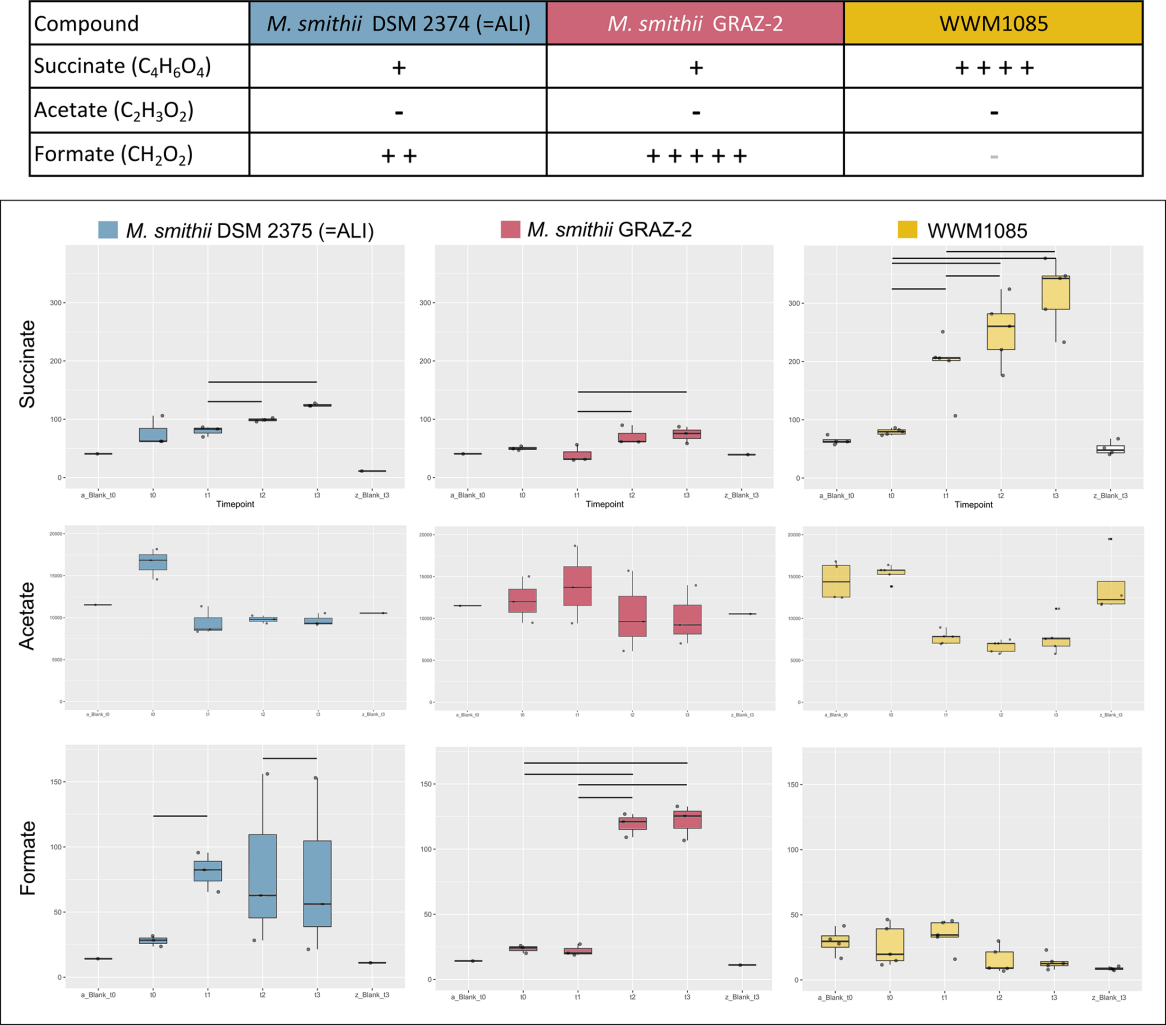
Metabolic dynamics and variability in various compounds among the studied *Methanobrevibacter* strains in MS medium supplemented with yeast extract. Concentrations in µM l^−1^. Upper panel (table): The ‘+’ indicate statistically significant changes over time, with the number of ‘+’ reflecting the fold change (e.g. 5 symbols denote a fivefold change or more). The grey ‘−’ signifies a statistical trend (*P* = 0.05 X). The symbol ‘−’ denotes no change. *Lower panel*: Boxplots of the respective measurements (all original data provided in Table S5). It is noteworthy that media blanks did not exhibit any statistically significant changes in compound levels.

All strains exhibited a notable and expected statistically significant uptake of acetate and production of succinate, which was highest in the WWM1085 culture (fourfold increase; Fig. 4) (Table S5).

Unlike the *M. smithii* cultures, WWM1085 did not exhibit formate accumulation in the medium. Specifically, there was a notable and substantial increase in formate accumulation for *M. smithii* GRAZ-2, reaching a fivefold increase (Fig. 4).

All biological properties of the tested isolates, including *M. smithii* DSM 861 (=PS^T^) are provided in Table 2.

### Description of *Methanobrevibacter intestini* sp. nov

*Methanobrevibacter intestini* (in.tes.ti’ni. L. gen. n. *intestini*, of the gut).

Coccobacillus with slightly tapered or rounded ends, measuring 0.16–0.43 µm in width and 0.29–0.54 µm in length, occurring mostly in pairs or short chains. Strictly anaerobic. Grows and produces methane from H_2_ and CO_2_. Growth is dependent on the presence of acetate and additional organics such as yeast extract. Formate cannot serve as the sole electron donor for growth. No growth on ethanol or methanol. The optimum growth temperature is 35–39 °C (minimum: 30 °C, maximum: 40 °C), and the optimum pH is 5.5–7.5 (minimum: 6.5, maximum: 10). The major lipid is archaeol, followed by caldarchaeol and cyclic archaeol. Lipid-associated sugars detected include glucose, fructose, rhamnose, ribose and xylose.

The type strain is WWM1085^T^ (=DSM 116060^T^, CECT 30992^T^), isolated from human faeces from an individual in the United States.

The DNA G+C content of strain WWM1085 is 30.30 mol%.

The draft genome and the 16S rRNA gene sequence of the type strain are available under GenBank accession numbers NQLD00000000 and PQ686616, respectively.

## Supplementary material

10.1099/ijsem.0.006751 Uncited Fig. S1.

10.1099/ijsem.0.006751 Uncited Supplementary Material 1.

## Abbreviations

AAI: amino acid identity
ANI: average nucleotide identity
FACS: fluorescence-activated cell sorting
MAGs: metagenome-assembled genomes
NMR: nuclear magnetic resonance
