# Supplementary material for: Expanding the cultivable human archaeome: Methanobrevibacter intestini sp. nov. and strain Methanobrevibacter smithii ‘GRAZ-2’ from human faeces
Source: Int J Syst Evol Microbiol. 2025 Apr 16;75(4):006751. doi: 10.1099/ijsem.0.006751 (PMC12281867; doi:10.1099/ijsem.0.006751)
Supplement: Uncited Fig. S1. [file ijsem-75-06751-s001.pdf]

Supplementary Information

Expanding the cultivable human archaeome: *Methanobrevibacter intestini* sp. nov. and strain *Methanobrevibacter smithii* "GRAZ-2" from human feces

Viktoria Weinberger[1], Rokhsareh Mohammadzadeh[1], Marcus Blohs[1], Kerstin Kalt[1], Alexander Mahnert[1], Sarah Moser[1], Marina Cecovini[1], Polona Mertelj[1], Tamara Zurabishvili[1], Bhawna Arora[1] , Jacqueline Wolf[3], Tejus Shinde[1], Tobias Madl[2,4], Hansjörg Habisch[4], Dagmar Kolb[5,6], Dominique Pernitsch[5], Kerstin Hingerl[5], William Metcalf[7], Christine Moissl-Eichinger[1,2*]

[1] D&R Institute of Hygiene, Microbiology and Environmental Medicine, Medical University of Graz, Graz, Austria
[2] BioTechMed Graz, Graz, Austria
[3] Research Group Metabolomics, Leibniz Institute DSMZ-German Collection of Microorganisms and Cell Cultures GmbH, Braunschweig, Germany.
[4] Otto Loewi Research Center, Medicinal Chemistry, Research Unit Integrative Structural Biology, Medical University of Graz, Graz, Austria
[5] Core Facility Ultrastructure Analysis, Medical University of Graz, Graz, Austria
[6] Gottfried Schatz Research Center, Cell Biology, Histology and Embryology, Medical University of Graz, Graz, Austria
[7] Department of Microbiology, University of Illinois, Urbana, Illinois, USA

*Corresponding author
christine.moissl-eichinger@medunigraz.at

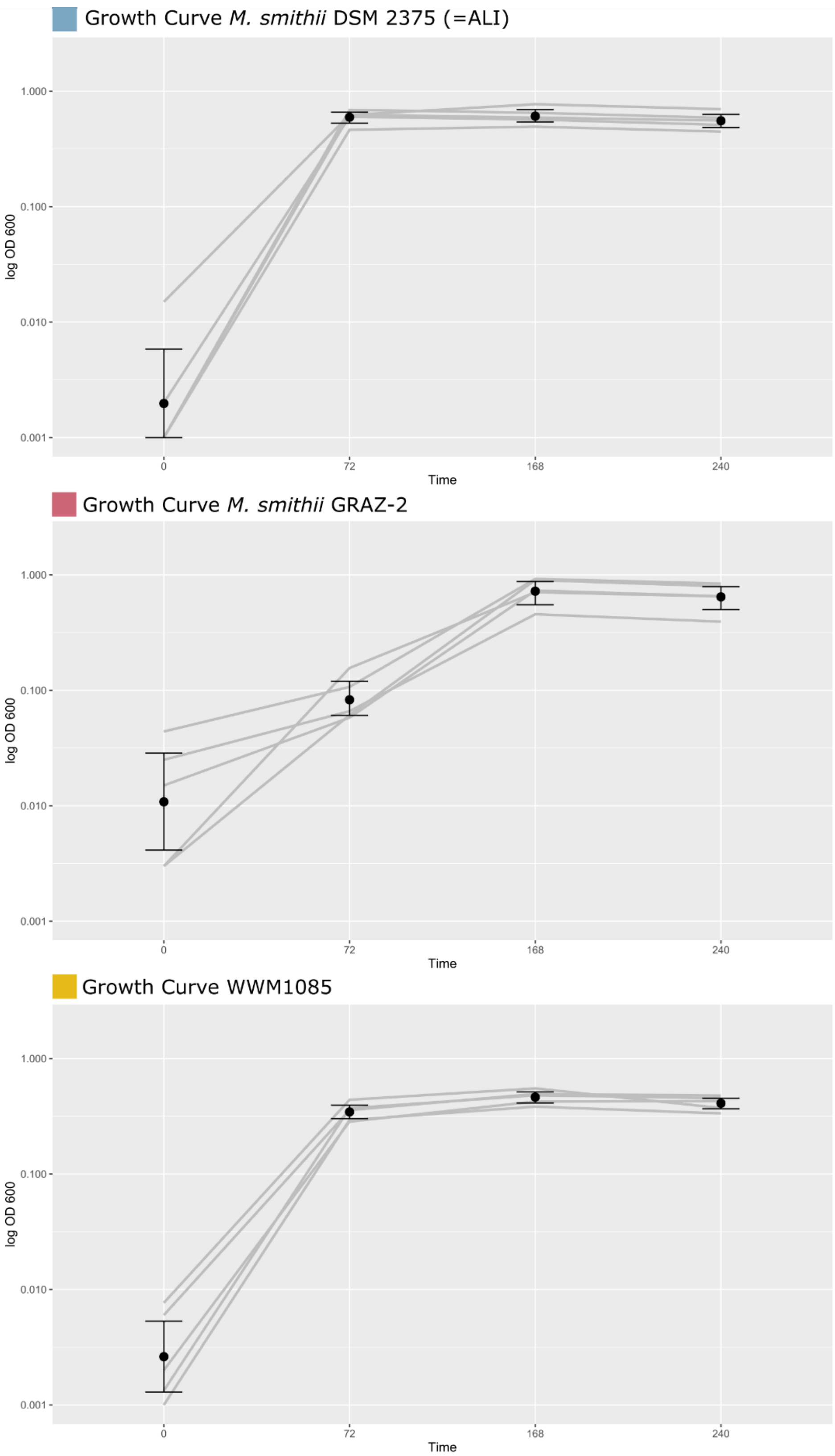

Supplementary Fig. S1: Growth curves. X-axis indicates time in hours, y-axis displays the log OD_{600}. *M. smithii* DSM 2375 (=ALI) and WWM1085 reached the stationary phase at 72 h, while *M. smithii* GRAZ-2 reached the stationary phase after 168 h.
